# Short‐term blood pressure changes have a more strong impact on stroke and its subtypes than long‐term blood pressure changes

**DOI:** 10.1002/clc.23242

**Published:** 2019-07-30

**Authors:** Rongrong Guo, Yanxia Xie, Jia Zheng, Yali Wang, Yue Dai, Zhaoqing Sun, Liying Xing, Xingang Zhang, Yingxian Sun, Liqiang Zheng

**Affiliations:** ^1^ Department of Clinical Epidemiology, Library Shengjing Hospital of China Medical University Shenyang P. R. China; ^2^ Department of Cardiology Shengjing Hospital of China Medical University Shenyang P. R. China; ^3^ Institute of Chronic Disease Liaoning Provincial Center for Disease Control and Prevention Shenyang P. R. China; ^4^ Department of Cardiology The First Affiliated Hospital of China Medical University Shenyang P. R. China

**Keywords:** blood pressure, blood pressure changes, cohort study, stroke

## Abstract

**Background:**

Elevated blood pressure (BP) is closely related to stroke and its subtypes. However, different time periods changes in BP may result in differential risk of stroke.

**Hypothesis:**

Short‐term blood pressure changes have a more strong impact on stroke and its subtypes than long‐term blood pressure changes.

**Methods:**

We designed the study on the effects of short‐ (2008‐2010) and long‐term (2004‐2010) BP changes on stroke events (2011‐2017), including 22 842 and 28 456 subjects, respectively. The difference in *β* coefficients between short‐ and long‐term BP changes on the effects of stroke were examined using the Fisher Z test.

**Results:**

During a median 12.5‐year follow‐up period, 1014 and 1505 strokes occurred in short‐ and long‐term groups. In short‐term group, going from prehypertension to hypertension, the risk of stroke events increased (stroke: hazard ratio [HR] = 1.537 [1.248‐1.894], ischemic stroke: 1.456 [1.134‐1.870] and hemorrhagic stroke: 1.630 [1.099‐2.415]); going from hypertension to prehypertension, the risk of stroke events decreased (stroke:0.757 [0.619‐0.927] and hemorrhagic stroke:0.569 [0.388‐0.835]). Similarly, in long‐term group, going from prehypertension to hypertension, individuals had an increased risk of stroke (1.291, 1.062‐1.569) and hemorrhagic stroke (1.818, 1.261‐2.623); going from hypertension to prehypertension, participants had a decreased risk of stroke (0.825, 0.707‐0.963) and hemorrhagic stroke (0.777, 0.575‐0.949). Furthermore, the effects of BP changes during short‐term period on stroke events were greater than that in long‐term period.

**Conclusions:**

Short‐ and long‐terms BP changes were both associated with the risk of stroke events. Furthermore, short‐term BP changes had a stronger impact than did long‐term changes on risk of stroke events.

## 1 INTRODUCTION

Stroke is the leading cause of death in China,^1^, ^2^ and high blood pressure (BP) is a major risk factor for stroke.^3^ Worldwide, approximately 54% of strokes are attributable to hypertension.^4^ Hence, lowering BP can effectively reduce the incidence of stroke. In the past decade or so, the prevalence of hypertension in rural areas of northeast China has changed considerably,^5^, ^6^ and the impact of this rising trend on the incidence, mortality of stroke is worrisome.

In addition, previous traditional studies have been linked to the association of BP values at one point in time with cardiovascular disease (CVD) risk. However, intraindividual BP fluctuates dynamically over time, and measurements at a single point in time may not effectively predict disease risk, to achieve early intervention.^7^ Gosmanova et al determined that traditional analysis would underestimate the true relationship between elevated BP levels and disease, as a result of a “regression dilution” effect.^7^ Vascular damage leading to stroke is a complex dynamic process. Dynamic BP changes include more critical information for prediction of stroke risk, having higher accuracy and sensitivity.^8^ Several lines of evidence that visit‐to‐visit variability (VVV) of BP or trajectory changes in BP are associated with a higher incidence of CVD^9^, ^10^, ^11^, ^12^ and a higher risk of mortality.^13^, ^14^, ^15^ However, few studies have concentrated on the effects of BP changes on stroke and its subtypes, and most of those studies were conducted either in Western countries^7^, ^10^ or focused on the adverse outcomes affected by BP changes during the same time period^16^, ^17^ or same multiple time periods.^9^ There is rare evidence of the effects of short‐ and long‐term BP changes on stroke and its subtypes in the general population, while the effects of long‐ and short‐term BP changes may be different.

We hypothesized that BP changes will affect to stroke and its subtypes according to three aspects: first, baseline BP levels are positively correlated with the risk of stroke for the same degree of BP changes; second, the direction of BP change (increase or decrease) is an important aspect of responding to BP changes; third, the rate of BP changes (high‐increasing or low‐increasing, high‐decreasing or low‐decreasing) have different effects on stroke in the same BP level changes. Therefore, we aimed to analyze and compare the effects of same level BP changes over a short‐ and long‐term period on the risk of stroke and its subtypes.

## 2 METHODS

### 2.1 Study population

We carried out a large‐scale epidemiological follow‐up study.^18^, ^19^ Briefly, from 2004 to 2006, a multistage, random cluster sampling process was used to select a representative sample aged ≥35 years in rural areas of Fuxin County in Liaoning Province, China. All study participants were invited to return for follow‐up: from January to July 2008 (follow‐up 1); from July to December 2010 (follow‐up 2); and from March to December 2017 (follow‐up 3). The study population inclusion and exclusion process is illustrated in Figure S1. Of the 45 925 participants at baseline, 3883 subjects had missing contact information or refused to attend the follow‐up, and 42 042 (91.5%) participants were eligible to attend the follow‐up at least one time.

The study protocol was approved by the China Medical University Research Ethics Committee, and written informed consent was obtained from all subjects or their caregivers.

### 2.2 Study design

**Figure 1:**
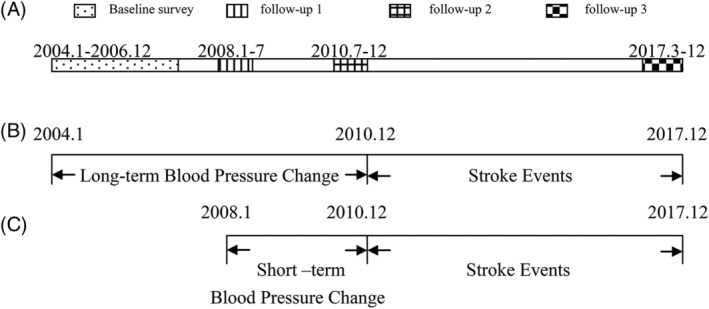
Study designs used for analysis of the associations between short‐ and long‐term blood pressure changes and stroke events. Description of baseline and three follow‐up surveys A. Effects of short‐term B, and long‐term C, blood pressure changes on stroke events

We have completed a baseline survey and three follow‐up surveys (Figure 1A). To investigate the effects of BP changes over short‐ and long‐term time intervals on stroke events, we collected new cases of stroke during the same time period between the short‐ and long‐term groups. Therefore, the study designs used for analysis of the associations between long‐ (from January 2004 to December 2010) and short‐term (from January 2008 to December 2010) BP changes and stroke events is shown in Figure 1B,C.

### 2.3 Data collection and measurements

Using a standardized questionnaire to collect demographic variables (gender, age, and ethnicity), lifestyle factors (current smoking, current drinking status, and physical activity), history of disease (stroke, coronary heart disease [CHD], family history of hypertension, diabetes, or hyperlipidemia), and information on antihypertensive medications.^18^, ^19^

When measuring weight and height, subjects did not wear coats and shoes. Body mass index (BMI) was calculated as the weight divided by the height squared (kg/m^2^). BP measurements were performed using a standard electronic automated sphygmomanometer (HEM‐741C; Omron, Tokyo, Japan). A trained and certified observer used an American Heart Association protocol to perform BP measurements.^20^ Participants were advised to avoid alcohol consumption, cigarette smoking, drinking coffee/tea, and exercise for at least 30 minutes, and to rest at least 5 minutes before BP measurement. The individual's BP value used for analyses was the average of three BP measurements. According to the JNC7 criteria,^21^ subjects were categorized as having normotension (SBP < 120 and DBP < 80 mm Hg), prehypertension (120 ≤SBP  < 140 mm Hg or 80 ≤ DBP < 90 mm Hg) or hypertension (SBP/DBP ≥ 140/90 mm Hg or receiving antihypertensive treatment). The subject's BP classifications may change from one classification to another or maintain stabilization. All subjects were divided into nine categories according to the BP classifications changes before and after.

### 2.4 Study outcomes

The present study outcome was stroke events, including ischemic stroke, hemorrhage stroke and uncategorized stroke. Stroke events were confirmed according to the WHO Multinational Monitoring of Trends and Determinants in Cardiovascular Disease (MONICA) criteria: a sudden onset of focal (or global) disturbance of cerebral function lasting more than 24 hours (unless interrupted by surgery or death) with no apparent nonvascular cause.^22^ The classification of ischemic or hemorrhagic was done on the basis of clinical presentation, and confirmation by CT or MRI was required, with reference to the MONICA criteria. The information about the main outcome was collected by qualified investigators every regular period of time (regular every 3 months). The relevant information about new stroke cases was obtained by direct reference to medical records. All materials obtained by investigators were independently reviewed by the Endpoint Evaluation Committee.

### 2.5 Statistical methods

Data were presented as the means ± SD, and number (percentages). The rates of events were presented as the number of events per 1000 person‐years. The proportional hazards assumption of the Cox proportional hazards models was confirmed using Schoenfeld residuals. We used multivariable Cox proportional hazards models to calculate the hazard ratio (HR) with 95% confidence intervals (CIs) for the associations between BP categories and stroke events, adjusted for age, gender, ethnicity, SBP, DBP, BMI, current smoking, current drinking, physical activity, education level, antihypertensive treatment, family history of hypertension, history of diabetes and hyperlipidemia, and the duration of hypertension. All analyses were performed using SPSS 22.0 (IBM Inc., Chicago, Illinois). The difference in *β* coefficients between short‐ and long‐term groups derived from the Z value was examined using the Fisher Z test.^23^ *P* < .05 was considered statistically significant.

## 3 RESULTS

Our results included 22 842 short‐term group and 28 456 long‐term group study population, of which 49.2% and 50.8% were women. The mean age were 51.54 (SD: 10.65) and 49.73 (SD: 10.91) years. Table 1 shows the mean values and percentages of study variables before BP changes for the two groups. In short‐ and long‐term groups, the mean values of SBP were 130.62 (SD: 14.34) mm Hg and 132.20 (SD: 21.07) mm Hg, the mean values of DBP were 81.12 (SD: 9.76) mm Hg and 81.71 (SD: 12.06) mm Hg, respectively. In addition, in the short‐ and long‐term groups, there were statistical significant differences in age, BMI, SBP, and DBP, gender, education level, physical activity, current smoking, current drinking, family history of hypertension, history of hyperlipidemia, antihypertensive treatment, and BP classification (all *P* < .05), except for ethnicity and history of diabetes.

**Table 1:** Baseline characteristics of short‐ and long‐term blood pressure changes groups

>Characteristics	>Short‐term BP changes group (n = 22 842)	>Long‐term BP changes group (n = 28 456)	>*t* or *χ* ^*2*^	>*P‐*value
Women, n (%)	11 246 (49.2)	14 450 (50.8)	12.12	<.001
Age, mean (SD), year	51.54 (10.65)	49.73 (10.91)	18.84	<.001
Ethnicity, n (%)			0.38	.827
Han populations	17 677 (77.4)	22 086 (77.6)		
Mongolian	4863 (21.3)	6000 (21.1)		
Others	302 (1.3)	370 (1.3)		
BMI, mean (SD), kg/m^2^	23.63 (2.48)	23.19 (2.72)	18.71	<.001
SBP, mean (SD), mmHg	130.62 (14.34)	132.20 (21.07)	−9.67	<.001
DBP, mean( SD), mmHg	81.12 (9.76)	81.71 (12.06)	−5.95	<.001
Education level, n (%)			25.20	<.001
Never or <5 years	9086 (39.8)	11 922 (41.9)		
Primary school	12 423 (54.4)	14 858 (52.2)		
Tertiary high school or higher education	1333 (5.8)	1676 (5.9)		
Physical activity, n (%)			13.08	.001
Low	5640 (24.7)	7397 (26.0)		
Moderate	10 606(46.4)	13 122 (46.1)		
High	6596 (28.9)	7937 (27.9)		
Current drinking, n (%)	8413 (36.8)	9091 (31.9)	27 391.25	<.001
Current smoking, n (%)	9151 (40.1)	11 808 (41.5)	27 553.65	<.001
Family history of hypertension, n (%)	2611 (11.4)	3542 (12.4)	12.41	<.001
History of diabetes, n (%)	72 (0.3)	98(0.3)	0.33	.568
History of hyperlipidemia, n (%)	428 (1.9)	694 (2.4)	18.91	<.001
Antihypertensive treatment, n (%)	1836 (8.0)	1732 (6.1)	74.54	<.001
Blood pressure classification, n (%)			464.45	<.001
Normotension	2948 (12.9)	5336 (18.8)		
Prehypertension	12 738 (55.8)	13 496 (47.4)		
Hypertension	7156 (31.3)	9624 (33.8)		

*Note*: Short‐term BP Changes Group: participants with short‐term (2008.1‐2010.12) blood pressure changes, number (percentage) and mean (SD) represent characteristics in follow‐up 1 (2008.01‐07); Long‐term BP Changes Group: participants with long‐term (2004.1‐2010.12) blood pressure changes, number (percentage) and mean (SD) represent characteristics in baseline survey (2004.01‐2006.12).

Abbreviations: BP, blood pressure; BMI, body mass index; DBP, diastolic blood pressure; SBP, systolic blood pressure.

**Figure 2:**
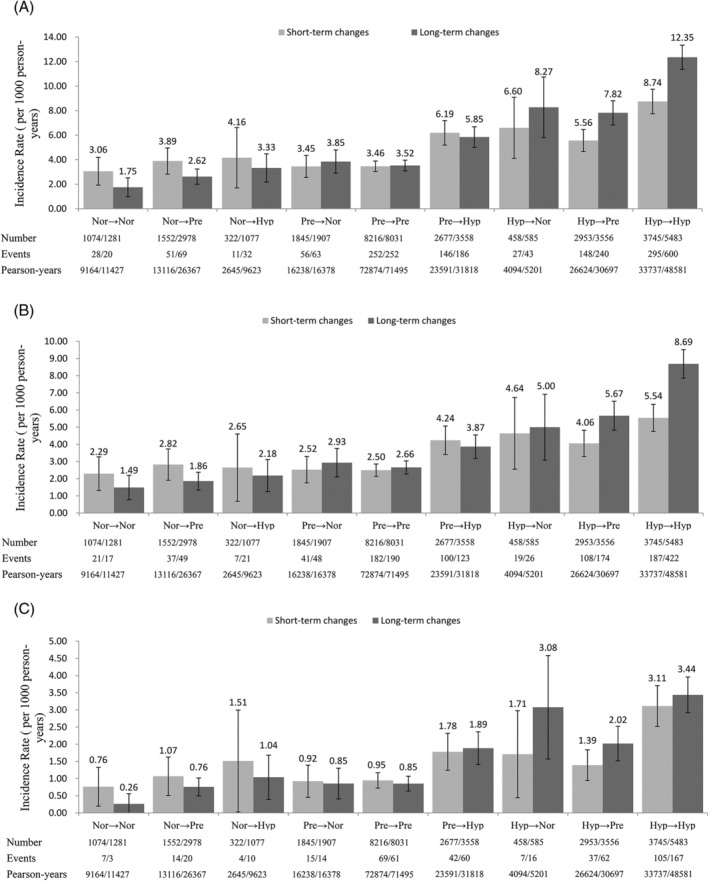
The incidence of stroke and its subtypes with short‐term and long‐term blood pressure changes. Stroke, A; ischemic stroke, B; hemorrhagic stroke, C; Nor, normotension; Pre, prehypertension; Hyp, hypertension. Error bars represent 95% CI

Figure 2 shows the incidence of stroke, ischemic stroke and hemorrhagic stroke in short‐ and long‐term groups. During a median follow‐up period of 12.5 years, we identified 1014 (702 ischemic, 300 hemorrhagic and 13 uncategorized) and 1505 (1070 ischemic, 413 hemorrhagic and 23 uncategorized) strokes during the periods of short‐ and long‐term BP groups, respectively. At the overall person‐years of 202 085 in short‐term group and 251 587 in long‐term group, the overall incidence of stroke was respectively 5.02/1000 and 5.98/1000 person‐years, ischemic stroke 3.47/1000 and 4.25/1000 person‐years, and hemorrhagic stroke 1.48/1000 and 1.64/1000 person‐years. In terms of the overall trend, subjects who were able to maintain normotension or decrease their BP to normotension had a lower incidence rate in short‐ and long‐term groups. Meanwhile, we found that normotensive subjects in short‐term group presented higher incidence rate of the three outcomes than long‐term group, no matter if they remained normotensive or became prehypertensive or hypertensive. While the contrary happen when considering hypertensive subjects in long‐term vs short‐term group. Furthermore, after short‐ and long‐term BP changes, the incidence rate of all the other eight categories was higher than that for maintaining the normotension.

Table 2 shows HRs and 95% CI for the association between short‐ and long‐term BP changes and risk of stroke events using Cox proportional hazards models. Individuals who changed from a state of prehypertension to hypertension during a short‐term time had an increased risk of stroke (HR = 1.537, 95% CI: 1.248‐1.894), ischemic stroke (HR = 1.456, 95% CI: 1.134‐1.870) and hemorrhagic stroke (HR = 1.630, 95% CI: 1.099‐2.415), compared with subjects who maintained prehypertension. In contrast, individuals who changed from hypertension to prehypertension during a short‐term time was showed a protective effect against stroke (HR = 0.757, 95% CI: 0.619‐0.927) and hemorrhagic stroke (HR = 0.569, 95% CI: 0.388‐0.835) compared with subjects who maintained hypertension. Similarly, individuals who changed from prehypertension to hypertension during a long‐term time had an increased risk of stroke (HR = 1.291, 95% CI: 1.062‐1.569) and hemorrhagic stroke (HR = 1.818, 95% CI: 1.261‐2.623), compared with subjects who maintained prehypertension. However, individuals who changed from hypertension to prehypertension during a long‐term time showed a protective effect against stroke (HR = 0.825, 95% CI: 0.707‐0.963) and hemorrhagic stroke (HR = 0.777, 95% CI: 0.575‐0.949), compared with subjects who maintained hypertension. The results of the sensitivity analysis (excluding antihypertensive treatment) were similar to the whole population in short‐ and long‐term groups **(** Table S1). In addition, *β* coefficients from Cox proportional hazards models were used to compare the effects of short‐ and long‐term BP changes on the risk of stroke and hemorrhagic stroke. For individuals whose BP changed from prehypertension to hypertension, short‐term changes were more strongly associated with increased risk of stroke than were long‐term changes on the basis of *β* coefficients (*β* = 0.430 vs 0.255, *P <* .001). Similarly, for individuals whose BP changed from hypertension to prehypertension, the *β* coefficient of short‐term BP changes was also higher compared to long‐term (*β* = −0.278 vs −0.193, *P <* .001). The opposite occurred in hemorrhagic stroke: for individuals whose BP changed from prehypertension to hypertension, long‐term changes were more strongly related to increased risk of hemorrhagic stroke compared to short‐term changes on the basis of β coefficients (β = 0.598 vs 0.488, *P <* .001). Conversely, for individuals whose BP changed from hypertension to prehypertension, the *β* coefficient of short‐term BP changes was higher compared to long‐term (*β* = −0.564 vs −0.253, *P <* .001).

**Table 2:** Survival analysis of long‐ and short‐term changes in blood pressure on stroke events^a^

>Changes in BP category	>Number	>Stroke			>Ischemic stroke		>Hemorrhagic stroke		
		>Hazard ratio (95% CI)	>*P‐* values	>*β*	>Hazard ratio (95% CI)	>*P*‐ values	>Hazard ratio (95% CI)	>*P* ‐values	>*β*
Short‐term BP changes group									
Normotension → normotension	1074	1.000 (Ref.)			1.000 (Ref.)		1.000 (Ref.)		
Normotension → prehypertension	1552	1.238 (0.761,2.015)	0.390	…	1.218 (0.687,2.160)	0.500	1.323 (0.519,3.371)	0.558	…
Normotension →hHypertension	322	1.223(0.595,2.513)	0.584	…	1.044 (0.431,2.529)	0.925	1.826 (0.519,6.430)	0.348	…
Prehypertension → prehypertension	8216	1.000 (Ref.)			1.000 (Ref.)		1.000 (Ref.)		
Prehypertension → normotension	1845	1.240(0.924,1.666)	0.152	…	1.291(0.914,1.824)	0.147	1.167(0.661,2.059)	0.595	…
Prehypertension → hypertension	2677	1.537 (1.248,1.894)	**<0.001**	0.430^b^	1.456 (1.134,1.870)	**0.003**	1.630 (1.099,2.415)	**0.015**	0.488^c^
Hypertension → hypertension	3745	1.000 (Ref.)			1.000 (Ref.)		1.000 (Ref.)		
Hypertension → normotension	458	1.077 (0.719,1.613)	0.720	…	1.151 (0.709,1.868)	0.570	0.887 (0.406,1.941)	0.765	…
Hypertension → prehypertension	2953	0.757(0.619,0.927)	**0.007**	−0.278^2^	0.836(0.656,1.066)	0.149	0.569(0.388,0.835)	**0.004**	−0.564^c^
Long‐term BP changes group									
Normotension → normotension	1281	1.000 (Ref.)			1.000 (Ref.)		1.000 (Ref.)		
Normotension → prehypertension	2978	1.299 (0.779,2.164)	0.316	…	1.118 (0.633,1.975)	0.701	2.332 (0.678,8.018)	0.179	…
Normotension → hypertension	1077	1.276 (0.714,2.279)	0.411	…	1.038 (0.533,2.019)	0.913	2.280 (0.602,8.638)	0.225	…
Prehypertension →prehypertension	8031	1.000 (Ref.)			1.000 (Ref.)		1.000 (Ref.)		
Prehypertension → normotension	1907	1.381(1.043,1.828)	**0.024**	…	1.390(1.008,1.917)	**0.045**	1.291(0.716,2.327)	0.396	…
Prehypertension → hypertension	3558	1.291 (1.062,1.569)	**0.010**	0.255^b^	1.114 (0.882,1.406)	0.367	1.818 (1.261,2.623)	**0.001**	0.598^c^
Hypertension → hypertension	5483	1.000 (Ref.)			1.000 (Ref.)		1.000 (Ref.)		
Hypertension → normotension	585	0.972 (0.709,1.331)	0.857	…	0.840 (0.562,1.255)	0.394	1.352 (0.800,2.285)	0.261	…
Hypertension → prehypertension	3556	0.825 (0.707,0.963)	**0.015**	−0.193^b^	0.846 (0.704,1.015)	0.072	0.777 (0.575,0.949)	**0.040**	−0.253^c^

*Note*: Short‐term blood pressure changes group, from the follow‐up 1 (2008.01‐2008.07) to the follow‐up 2 (2010.07‐2010.12); Long‐term blood pressure changes group, from the baseline surveys (2004.01–2006.12) to the follow‐up 2 (2010.07‐2010.12); Normotension, subjects with BP <120/80 mm Hg; prehypertension, subjects with BP of 120‐139/80‐89 mm Hg; hypertension, subjects with BP≥140/90 mmHg or antihypertensive treatment.

Abbreviations: BP, blood pressure; BMI, body mass index; CI, confidence interval; DBP, diastolic blood pressure; SBP, systolic blood pressure.

a Adjusted for variables in short‐ and long‐term groups, respectively: age, gender, ethnicity, SBP, DBP, BMI, education level, physical activity, current drinking, current smoking, family history of hypertension, history of diabetes, history of hyperlipidemia, antihypertensive treatment, the duration of hypertension.

b *β* Coefficients different from 0 for short‐ and long‐term blood pressure changes groups in stroke: *P* < 0.05.

c *β* Coefficients different from 0 for short‐ and long‐term blood pressure changes groups in hemorrhagic stroke: *P* < 0.05.

Bold values in Table 2 was considered statistically significant (*P* < .05).

**Figure 3:**
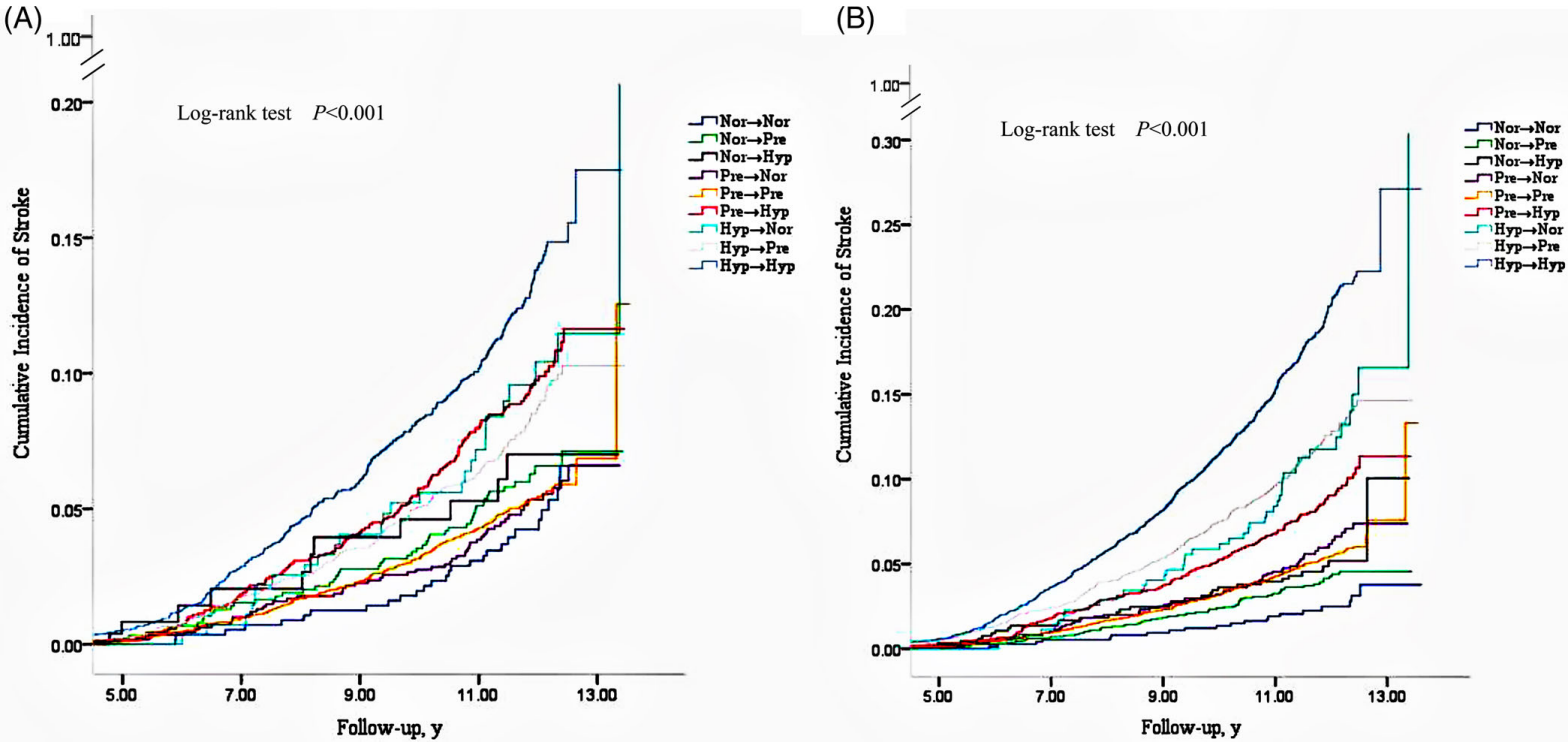
The cumulative incidence of stroke with short‐term and long‐term blood pressure changes. Short‐term blood pressure changes, A; long‐term blood pressure changes, B; Nor, normotension; Pre, prehypertension; Hyp, hypertension

Figure 3 shows the cumulative incidence risk of long‐ and short‐term BP changes to stroke. Regardless of short‐ or long‐term BP changes, individuals who were able to maintain or to decrease their BP values had a lower cumulative incidence of stroke. In contrast, those who had higher initial BP values or an increase in their BP values had a higher of cumulative incidence of stroke. The cumulative incidence of stroke for all other eight categories was higher than that for maintaining the normotension, and individuals who maintained the hypertension and normotension had the highest and lowest cumulative incidence, respectively.

## 4 DISCUSSION

We studied and compared the effects of BP changes over a short‐ and long‐term period on stroke and its subtypes in a prospective cohort study in Chinese rural adults, and found that the majority of individuals remained stable or experienced a slight increase in BP values over time. In general, significant positive associations were observed for rising BP and higher risk of stroke events, compared with individuals remaining stable or showing a decrease in BP values. In addition, compared to the same increase in BP levels over a long‐term period, the short‐term increase in BP had a greater impact on the risk of stroke events.

Previous studies have confirmed and reinforced the importance of BP change by different forms for stroke. Limited prospective studies in the form of longitudinal elevated or decreased BP trajectories at several time points have emphasized that increase in BP is a major risk factor for adverse outcomes of CVD (including stroke) in China or Western countries.^9^, ^12^, ^13^, ^15^, ^24^, ^25^ Another form of BP changes is the visit‐to‐visit blood pressure variability, and studies have found that higher variability of BP or SBP is a powerful predictor of cardiovascular events independently of mean SBP or DBP, which has been more common in previous studies.^7^, ^11^, ^14^, ^26^, ^27^, ^28^ A large longitudinal study of U.S. veterans found that greater long‐term visit‐to‐visit SBP variability was associated with higher risk of subsequent stroke, independent of mean SBP, which is consistent with our findings.^7^ Furthermore, our study considered baseline BP values and the effect of antihypertensive treatment. A significant proportion of individuals with hypertension with decreased BP showed a decreased risk of stroke. However, even if BP values were decreased, the incidence of stroke for individuals with hypertension or prehypertension was still higher than for those with normotension. The results suggest that a reduction in BP may not fully reverse the effect of sustained hypertension earlier in life, which may be because of physiological changes in the vasculature, such as arterial stiffening, fibrosis, and calcification.^28^ This finding was comparable to that in a previous study of 9845 individuals from the Atherosclerosis Risk in Communities (ARIC) study in the United States.^9^ Even so, lowering BP values below the guidelines of treatment is still the most direct and effective way to decrease incidence of stroke.^27^

In addition, with the hypertension as reference, individuals who changed from hypertension to prehypertension decreased the risk of stroke events in short‐ and long‐term groups. However, there was no statistical significance in the risk of stroke events in BP classifications of individuals who changed from hypertension to normotension compared with maintained hypertension. This is maybe a power issue (fewer people make the changing from hypertension to normotension), or it could also imply a J‐curve effect in lowering BP.^29^ Although some of the results in our subgroup analysis have no statistical significance, they cannot be ignored. We still cannot deny that antihypertensive treatment can reduce the risk of the stroke events. Our results may be by chance because of the small number of cases and insufficient statistical power.

Comparison of the effects of short‐ or long‐term changes in BP on stroke outcomes is the first study to date. Our results mainly found that short‐term BP changes had a greater impact on stroke going from prehypertension to hypertension or vice versa. A pair of opposite results were obtained with hemorrhagic stroke, where it appeared that short‐term BP changes had a greater impact going from hypertension to prehypertension and long‐term BP changes have a greater impact from prehypertension to hypertension. There is no clear mechanism to explain this difference. Compared to BP long‐term changes, short‐term changes in BP may make the compliance of large elastic arteries worse, resulting in more severe arterial stiffness^30^ and more frequent abnormal autoregulation,^31^ or may increase inflammatory markers, damage vascular endothelial cells and cause endothelial dysfunction.^31^, ^32^ Although we cannot be certain of the reasons for one result that long‐term BP changes had a greater impact on hemorrhagic stroke, it seems likely that the small number of hemorrhagic stroke events (only 43 and 61 events occurred in short‐term and long‐term groups) after 9 classifications might explain the discrepancy vs Further studies are needed to elucidate this issue.

Cumulative BP load is related to the risk of stroke in a later time period. In short‐ and long‐term BP change analyses, we found that initial BP level at a higher BP classification or more rapid increase in BP was significantly correlated with the cumulative incidence of stroke. Our results support previous findings of observational studies, which suggested that increase in BP is detrimental to health. A study of seven diverse US cohort studies estimated how BP changes affect the lifetime risk for CVD and found that increases in BP showed a positive relationship with remaining lifetime risk for CVD.^10^ Prevention efforts should continue to emphasize the importance of lowering BP and avoiding or delaying the incidence of hypertension to reduce the risk of stroke. In addition, our results also showed the necessity for dynamic analysis. Multiple longitudinal BP measurements to some extent prevent bias in assessing the risk of stroke incident in a single BP measurement, which provides a comprehensive explanation for the effects of BP changes on stroke incidence.

The strengths of our study include the relatively large sample size and long period of follow‐up period. Some limitations should also be considered in this study. First, our research was only based on a rural group of people in Northeast China, which limits the generalization of our results. More diverse populations are needed to confirm our results. Second, we did not have sufficient information on laboratory measurements, such as serum cholesterol, glucose, and inflammation biomarkers to control for other potential confounders. Third, it is also worth noting that grouping classifications of change in BP might have obscured some of the individual variability in temporal changes in BP and might have resulted in attenuated. Forth, the relatively small sample size in the BP changing groups might have been biased the results. More study population would be expected to predict the associations between BP changes and the risk of stroke. Fifth, information on the antihypertensive treatment of hypertensions in our study is limited. More information about hypertension population with or without antihypertensive treatment is needed to as the potential confounders to confirm our findings.

In conclusion, our study found that short‐ and long‐term changes in BP differentially affect the subsequent risk of stroke events independent of baseline BP levels and that elevated BP would increase the future risk of stroke and its subtypes. Furthermore, decreased BP can lower the risk of incidence but does not reverse the persistent negative effects of exposure to elevated BP. Even so, great attention should be paid to increasing the rate of hypertension control in rural areas of China.

## CONFLICT OF INTEREST

The authors declare no potential conflict of interests.

## Supporting information


**TABLE S1** Sensitivity analysis of long‐ and short‐term changes in blood pressure on stroke events after excluding antihypertensive treatment*

**FIGURE S1** The study population inclusion and exclusion process
