# Supplementary material for: Short‐term blood pressure changes have a more strong impact on stroke and its subtypes than long‐term blood pressure changes
Source: Clin Cardiol. 2019 Jul 30;42(10):925–33. doi: 10.1002/clc.23242 (PMC6788570; doi:10.1002/clc.23242)
Supplement: Supplementary file 1 — TABLE S1 Sensitivity analysis of long‐ and short‐term changes in blood pressure on stroke events after excluding antihypertensive treatment* [file CLC-42-925-s001.docx]

**Supplementary Table 1.** Sensitivity analysis of long-term and short-term changes in blood pressure on stroke events after excluding antihypertensive treatment*****

|  |  | Stroke | | |  | Ischemic Stroke | |  | Hemorrhagic Stroke | | |
| --- | --- | --- | --- | --- | --- | --- | --- | --- | --- | --- | --- |
| Changes in BP category | Number | Hazard Ratio (95%CI) | *P* Values | β |  | Hazard Ratio (95%CI) | *P* Values |  | Hazard Ratio (95%CI) | *P* Values | β |
| **Short-term blood pressure changes group** | |  |  |  |  |  |  |  |  |  |  |
| ≤120/80→≤120/80 | 1074 | 1.000 (Ref.) |  |  |  | 1.000 (Ref.) |  |  | 1.000 (Ref.) |  |  |
| ≤120/80→130~139/80~89 | 1552 | 1.238(0.761,2.014) | 0.390 | … |  | 1.217(0.687,2.158) | 0.501 |  | 1.315(0.517,3.349) | 0.565 | … |
| ≤120/80→≥140/90 | 260 | 1.415(0.687,2.918) | 0.347 | … |  | 1.172(0.483,2.848) | 0.725 |  | 2.331(0.653,8.324) | 0.193 | … |
| 130~139/80~89→130~139/80~89 | 8216 | 1.000 (Ref.) |  |  |  | 1.000 (Ref.) |  |  | 1.000 (Ref.) |  |  |
| 130~139/80~89→≤120/80 | 1845 | 1.265(0.941,1.699) | 0.119 | … |  | 1.308(0.925,1.849) | 0.128 |  | 1.202(0.681,2.122) | 0.526 | … |
| 130~139/80~89→≥140/90 | 2162 | 1.502(1.203,1.876) | **<0.001** | 0.407^#^ |  | 1.402(1.072,1.835) | **0.014** |  | 1.700(1.131,2.555) | **0.011** | 0.531^$^ |
| ≥140/90→≥140/90 | 2087 | 1.000 (Ref.) |  |  |  | 1.000 (Ref.) |  |  | 1.000 (Ref.) |  |  |
| ≥140/90→≤120/80 | 352 | 1.073(0.670,1.717) | 0.771 | … |  | 1.083(0.608,1.931) | 0.786 |  | 0.942(0.394,2.249) | 0.892 | … |
| ≥140/90→130~139/80~89 | 2347 | 0.768(0.603,0.977) | **0.032** | -0.264^&^ |  | 0.897(0.674,1.195) | 0.458 |  | 0.464(0.287,0.751) | **0.002** | -0.768^£^ |
| **Long-term blood pressure changes group** | |  |  |  |  |  |  |  |  |  |  |
| ≤120/80→≤120/80 | 1281 | 1.000 (Ref.) |  |  |  | 1.000 (Ref.) |  |  | 1.000 (Ref.) |  |  |
| ≤120/80→130~139/80~89 | 2978 | 1.301(0.780,2.171) | 0.313 | … |  | 1.135(0.642,2.008) | 0.662 |  | 2.276(0.661,7.840) | 0.193 | … |
| ≤120/80→≥140/90 | 917 | 1.216(0.667,2.218) | 0.523 | … |  | 0.980(0.488,1.970) | 0.955 |  | 2.161(0.559,8.358) | 0.264 | … |
| 130~139/80~89→130~139/80~89 | 8031 | 1.000 (Ref.) |  |  |  | 1.000 (Ref.) |  |  | 1.000 (Ref.) |  |  |
| 130~139/80~89→≤120/80 | 1907 | 1.393(1.052,1.845) | **0.021** | … |  | 1.399(1.014,1.930) | **0.041** |  | 1.310(0.726,2.364) | 0.370 | … |
| 130~139/80~89→≥140/90 | 2820 | 1.307(1.063,1.608) | **0.011** | 0.268^#^ |  | 1.144(0.892,1.466) | 0.289 |  | 1.859(1.266,2.730) | **0.002** | 0.620^$^ |
| ≥140/90→≥140/90 | 2847 | 1.000 (Ref.) |  |  |  | 1.000 (Ref.) |  |  | 1.000 (Ref.) |  |  |
| ≥140/90→≤120/80 | 522 | 0.963(0.679,1.365) | 0.831 | … |  | 0.825(0.522,1.303) | 0.408 |  | 1.277(0.727,2.242) | 0.395 | … |
| ≥140/90→130~139/80~89 | 3092 | 0.792(0.657,0.954) | **0.014** | -0.234^&^ |  | 0.835(0.668,1.043) | 0.112 |  | 0.687(0.482,0.979) | **0.038** | -0.375^£^ |

Abbreviations: Short-term blood pressure changes group, from the follow-up 1 (2008.01-2008.07) to the follow-up 2 (2010.07-2010.12); Long-term blood pressure changes group, from the baseline surveys (2004.01-2006.12) to the follow-up 2 (2010.07-2010.12); BP, blood pressure.

*****adjusted for variables in short-term and long-term groups, respectively: age, gender, ethnicity, SBP (systolic blood pressure), DBP (diastolic blood pressure), BMI (body mass index), education level, physical activity, current drinking, current smoking, family history of hypertension, history of diabetes, history of hyperlipidemia, and , the duration of hypertension.

^#,&^ β Coefficients different from 0 for short-term and long-term blood pressure changes groups in stroke: *P* <0.05.

^$,£^ β Coefficients different from 0 for short-term and long-term blood pressure changes groups in hemorrhagic stroke: *P* <0.05.
