# Supplementary figures and images for: Short‐term blood pressure changes have a more strong impact on stroke and its subtypes than long‐term blood pressure changes
Source: Clin Cardiol. 2019 Jul 30;42(10):925–33. doi: 10.1002/clc.23242 (PMC6788570; doi:10.1002/clc.23242)

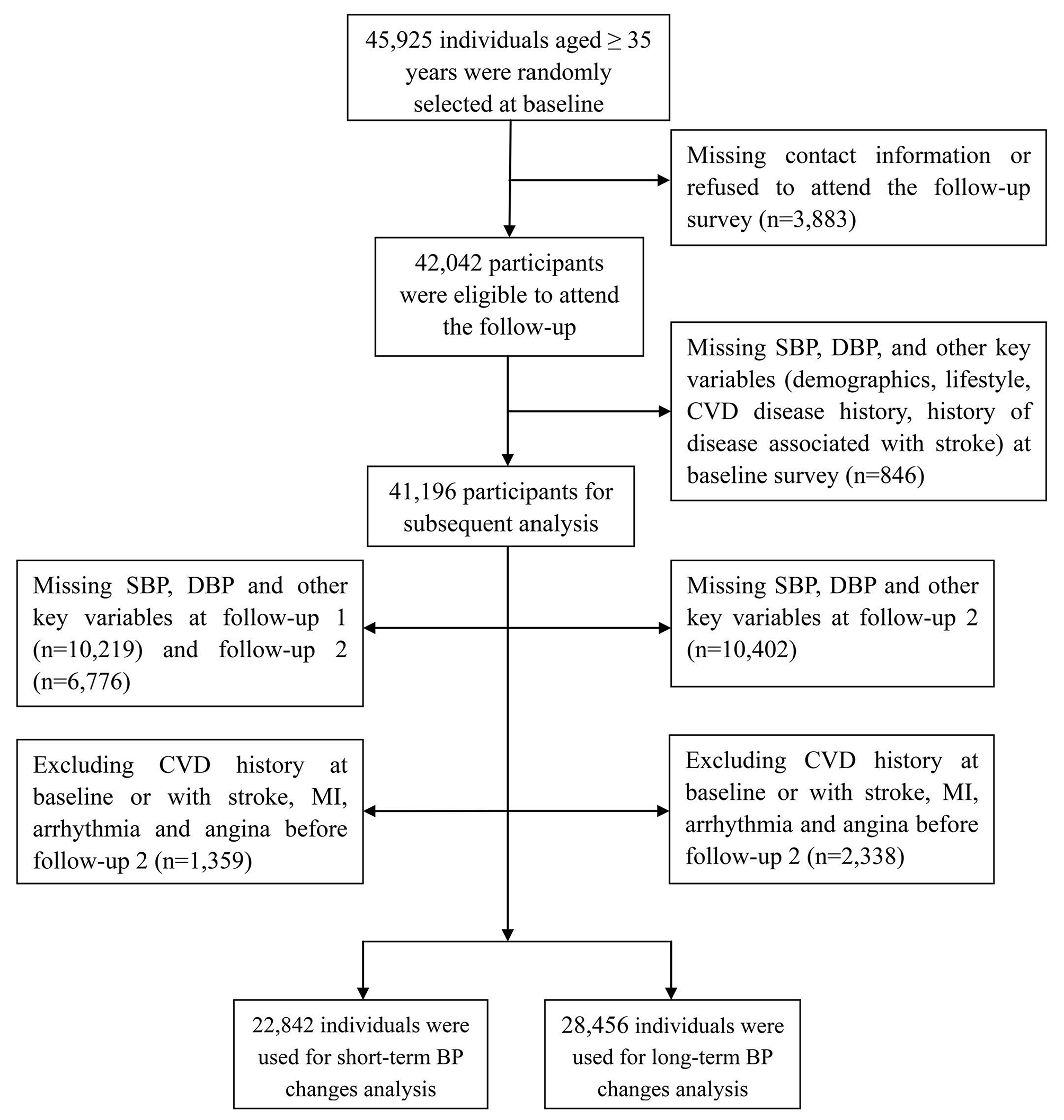

Supplement: Supplementary file 2 — FIGURE S1 The study population inclusion and exclusion process [file CLC-42-925-s002.tif]
